# Ischemic Vascular Complications in Early Systemic Sclerosis (SSc): A Longitudinal Inception Cohort Study of Associated Clinical Factors and Mortality

**DOI:** 10.3390/jcm15072575

**Published:** 2026-03-27

**Authors:** Suparaporn Wangkaew, Chammaliang Preecha, Narawudt Prasertwitayakij, Juntima Euathrongchit

**Affiliations:** 1 Division of Rheumatology, Department of Internal Medicine, Faculty of Medicine, Chiang Mai University, Chiang Mai 50200, Thailand; 2 Division of Cardiology, Department of Internal Medicine, Faculty of Medicine, Chiang Mai University, Chiang Mai 50200, Thailand; 3 Division of Diagnostic Radiology, Department of Radiology, Faculty of Medicine, Chiang Mai University, Chiang Mai 50200, Thailand

**Keywords:** systemic sclerosis, scleroderma, coronary artery disease, stroke, digital gangrene

## Abstract

**Background/Objectives:** Predictors of ischemic vascular complications (IVCs)—including coronary artery disease (CAD), ischemic stroke, and digital gangrene—in patients with early SSc remain insufficiently defined. Therefore, we aim to determine the incidence, risk factors, and mortality associated with IVCs in early SSc. **Methods:** An inception cohort of patients with early SSc at the Rheumatology Clinic, Maharaj Nakorn Chiang Mai Hospital, Thailand, was studied from January 2010 to December 2023. Clinical, laboratory, and cardiopulmonary assessments were performed at baseline and annually thereafter. **Results:** A total of 146 patients (83 female, 119 DcSSc) were enrolled, with a mean disease duration of 11.5 ± 8.9 months from the first non-Raynaud’s phenomenon (NRP). The mean follow-up was 8.0 ± 3.9 years. Seventeen patients (11.6%) developed IVCs, three CAD, four ischemic stroke, eight digital gangrene, and two digital gangrene plus CAD. The median time to first IVCs was two years. The overall incidence rate of IVCs from the NRP was 1.44 per 100 person-years (95% CI 0.89–2.32). Independent factors associated with IVCs included baseline (BL) digital ulcer, traumatic ulcer, LVEF < 50%, elevated pro-BNP, and any atrial fibrillation. BL pro-BNP and dyslipidemia were independently associated with CAD, whereas BL pro-BNP and any atrial fibrillation were associated with ischemic stroke. BL digital ulcer, traumatic ulcer, and any LVEF < 50% were associated with digital gangrene. All-cause mortality was higher among patients with IVCs than those without (9 [52.9%] vs. 37 [28.7], *p* = 0.043). **Conclusions:** In this study, IVCs were uncommon in early SSc, but were associated with increased mortality. Digital ulcers, traumatic ulcers, atrial fibrillation, impaired LVEF, and elevated pro-BNP identified the patients at higher risk of IVCs.

## 1. Introduction

Vasculopathy is a hallmark of systemic sclerosis (SSc), coexisting with immune dysregulation and inflammation in the early disease phase. These processes promote fibroblast proliferation and excessive collagen deposition in the skin and internal organs, leading to progressive organ dysfunction characteristic of SSc. Endothelial dysfunction, vascular injury, and fibro-occlusive vascular remodeling contribute to both microvascular and macrovascular complications, resulting in ischemic vascular complications presented with tissue ischemia and organ dysfunction [1].

Microvascular involvement manifests as Raynaud’s phenomenon, pulmonary arterial hypertension (PAH), scleroderma renal crisis (SRC), and microcoronary artery involvement. In contrast, macrovascular disease reflects accelerated atherosclerosis, clinically presenting as myocardial infarction (MI), ischemic stroke, and peripheral vascular disease (PVD). Proposed mechanisms underlying accelerated atherosclerosis in SSc include: (i) disease-specific factors such as chronic low-grade inflammation, endothelial autoantibodies, and increased vasospastic mediators; and (ii) traditional cardiovascular risk factors such as hypertension, diabetes mellitus, dyslipidemia, and smoking [2,3]. Whether SSc independently increases cardiovascular risk beyond traditional risk factors remains debated. A recent meta-analysis of 17 retrospective cohorts demonstrated significantly increased risk of MI, stroke, and PVD in SSc across heterogeneous populations, although the excess stroke risk was limited to non-Asian populations [4].

Reported risk factors for coronary artery disease (CAD) in SSc include male sex, older age, longer disease duration, PAH, and traditional atherosclerotic risk factors [5,6]. Corticosteroids, aspirin, NSAIDs, and Cox-2 inhibitors have also been implicated [7,8]. Ischemic stroke in SSc has been associated with atrial fibrillation, hypertension, diabetes mellitus, and aspirin, NSAIDs, or corticosteroid use [7,8]. Digital gangrene is a severe ischemic vascular complication associated with substantial morbidity. In the EUSTAR prevalence cohort, diffuse cutaneous SSc (DcSSc) and longer disease duration were major risk factors for digital gangrene [9]. Based on the prior literature, we specifically evaluated clinical manifestations, laboratory findings, cardiac parameters, and traditional cardiovascular risk factors as potential predictors of IVCs.

To our knowledge, data comprehensively evaluating the incidence, associated factors, and mortality of arterial ischemic vascular complications (IVCs), including coronary artery disease, ischemic stroke, and digital gangrene, with long-term follow-up in patients with early SSc remain limited. Therefore, the present study focuses specifically on clinically relevant arterial ischemic complications reflecting SSc-related vasculopathy. We aimed to: (i) determine the incidence of IVCs; (ii) compare baseline and cumulative clinical features between patients with and without these complications; (iii) identify factors associated with their development; and (iv) assess all-cause mortality.

## 2. Material and Methods

### 2.1. Study Design and Setting

This study was a sub-study of an inception cohort of early SSc conducted from January 2010 to December 2023 at Maharaj Nakorn Chiang Mai Hospital, a tertiary academic medical center in Chiang Mai, Thailand. The study was conducted in accordance with the Declaration of Helsinki and was approved by the Institutional Research Ethics Committee. All participants provided written informed consent prior to enrolment.

### 2.2. Study Population

All consecutive SSc patients ≥ 18 years with a disease duration of ≤3 years from the first non-Raynaud’s phenomenon (NRP) were enrolled. All patients fulfilled the 2013 ACR/EULAR classification criteria for SSc [10]. Patients were classified as diffuse cutaneous SSc (DcSSc) or limited cutaneous SSc (LcSSc) according to Medsger and LeRoy criteria [11]. Exclusion criteria included overlap syndromes such as rheumatoid arthritis [12], systemic lupus erythematosus (SLE) [13], mixed connective tissue disease [14], idiopathic inflammatory myopathies [15], Sjögren’s syndrome [16] and primary systemic vasculitis [17,18,19], based on established classification criteria and clinical evaluation by experienced rheumatologists. Systematic serological screening for all overlap conditions was not routinely performed. Additional exclusion criteria included fewer than one follow-up visit and pre-existing IVCs occurring before the diagnosis of SSc (defined as the time of the first NRP).

### 2.3. Data Collection

Clinical evaluation and laboratory investigations including complete blood count, creatinine, erythrocyte sedimentation rate (ESR), creatine kinase (CK), and pro-B-type natriuretic peptide (pro-BNP) were performed at baseline and at six-month intervals. Cardiopulmonary assessments, including electrocardiography (ECG), echocardiography, and high-resolution computed tomography (HRCT), were conducted at baseline and annually thereafter. Clinical manifestations, comorbidities, medications, organ complications, and investigations were recorded at baseline and longitudinally every six months. ECGs and echocardiography were interpreted by an experienced cardiologist (NP), and HRCT by an experienced chest radiologist (JE). IVCs were recorded at baseline and at each outpatient or inpatient visit. For patients with multiple IVCs, only the first event was included in time-to-event analyses.

**Definitions**

**Ischemic vascular complications (IVCs)** included (1) **CAD**, MI or atherosclerotic heart disease supported by a coronary angiography, and all were clinically adjudicated by a cardiologist; (2) **ischemic stroke** confirmed by computed tomography (CT) or magnetic resonance imaging (MRI) and managed by a neurologist; and (3) **digital gangrene**—irreversible ischemic necrosis of digits attributable to SSc-related vasculopathy. Alternative causes of digital necrosis, including embolic events, infection, primary systemic vasculitis, and antiphospholipid syndrome (APS), were clinically excluded based on comprehensive assessment by experienced rheumatologists, supported by laboratory investigations, autoantibody testing, and specialist evaluation when clinically indicated. APS was excluded based on compatible ischemic clinical features and presence of antiphospholipid antibodies, including lupus anticoagulant, anti-cardiolipin antibodies and anti-β2 glycoprotein I antibodies [20]. Systematic screening for inherited thrombophilia, such as factor V Leiden and prothrombin gene mutations, was not routinely performed.

**Traditional cardiovascular risk factors** were defined as follows: **hypertension**—systolic blood pressure ≥ 140 mmHg, diastolic blood pressure ≥ 90 mmHg, and on antihypertensive medication; **dyslipidemia**—diagnosed and receiving lipid-lowering treatment; **diabetes mellitus**—diagnosed and on treatment; and **smoking**—ever been a smoker. Interstitial lung disease (ILD) was defined by HRCT; suspected pulmonary hypertension (PH) was determined by echocardiography [21]. Additional definitions of SSc-related organ involvement and cumulative organ involvement followed definitions from a previous publication [22].

### 2.4. Statistical Analysis

Categorical variables are presented as frequencies and percentages, whereas continuous variables are expressed as mean ± standard deviation (SD) or median with interquartile range (IQR), as appropriate according to data distribution. Group comparisons were performed using the Chi-square test or Fisher’s exact test for categorical variables, and Student’s *t*-test or Mann–Whitney *U* test for continuous variables. Factors associated with overall IVCs and each subtype (CAD, ischemic stroke, digital gangrene) were identified using multivariable Cox regression with backward stepwise selection. Proportional hazards, concordance (Harrell’s C), and goodness-of-fit (Cox–Snell residuals) assessed model adequacy. Kaplan–Meier survival estimates from the first NRP were analyzed using the log-rank test. Due to the limited number of events, patients with simultaneous combined events were counted in each relevant category for subtype-specific time-to-event analyses. Analyses were performed using Stata version 14.0 (StataCorp, College Station, TX, USA), with *p*-value < 0.05 considered statistically significant.

## 3. Results

### 3.1. Baseline Characteristics

**Figure 1:**
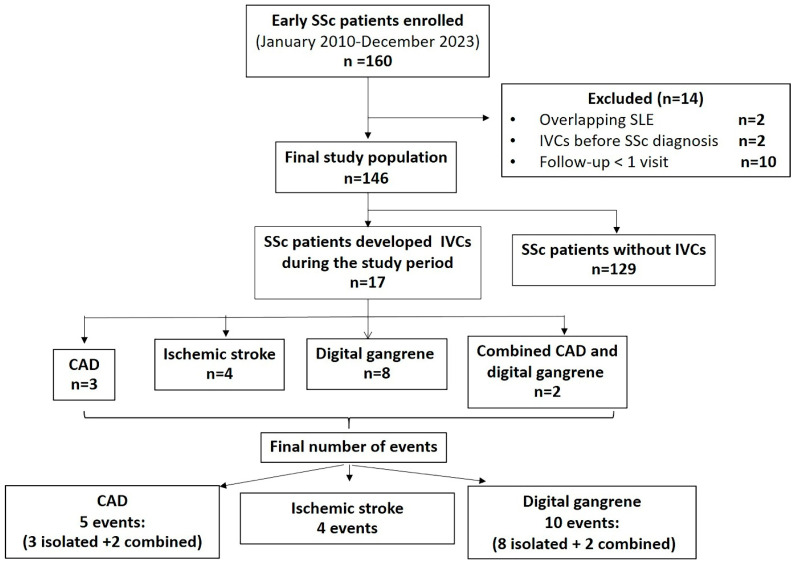
Flow diagram of the study population.

A total of 160 patients with early SSc were initially enrolled. Fourteen were excluded: two who later fulfilled criteria for SLE, two with a history of IVCs before the first NRP (one CAD and one ischemic stroke), and ten who had fewer than one follow-up visit. Thus, 146 patients were included in the final analysis. Of these, 83 (56.8%) were female, 119 (81.5%) had DcSSc, 113 (77.4%) were anti-topoisomerase I-positive, and 11 (7.5%) were anti-centromere-positive. No patients developed SRC during the study period. Mean ± SD age at the first NRP was 52.6 ± 9.4 years, and mean disease duration from the first NRP to cohort entry was 11.5 ± 8.9 months, and mean follow-up from the first NRP was 8.0 ± 3.9 years. During follow-up, 17 patients (11.6%) developed IVCs: three developed CAD, four ischemic stroke, eight digital gangrene, and two developed both CAD and digital gangrene simultaneously. For event-based analyses, the final numbers of events were five for CAD (three isolated and two combined), four for ischemic stroke, and ten for digital gangrene (eight isolated and two combined) (Figure 1).

### 3.2. Incidence Rate of Overall IVCs, CAD, Ischemic Stroke, and Digital Gangrene

The median (IQR) time from the first NRP to the first IVC was 2.0 (1, 8) years. Median times to first CAD, ischemic stroke, and digital gangrene were 2.0 (2, 8), 4.5 (1.2, 7.7), and 1.5 (0.9, 8.5) years, respectively. The overall incidence rate of IVCs was 1.44 per 100 person-years (95% CI 0.89–2.32). The incidence rates of CAD, ischemic stroke, and digital gangrene were 0.40 (95% CI 0.17–0.97), 0.32 (95% CI 0.12–0.86), and 0.83 (95% CI 0.45–1.55) per 100 person-years, respectively. Patients were categorized as those without IVCs (n = 129, 88.4%) and those with IVCs (n = 17, 11.6%).

### 3.3. Comparison of Clinical Manifestations and Investigations at Study Entry

Table 1 summarizes baseline clinical manifestations, laboratory findings, cardiopulmonary assessments, and medication profiles in patients with and without IVCs. Compared with the non-IVC group, patients who developed IVCs had a significantly higher prevalence of digital pitting scars, digital ulcers, traumatic ulcers, atrial fibrillation, left ventricular ejection fraction (LVEF) < 50%, and elevated serum CK and pro-BNP levels. No significant differences were observed between the two groups regarding demographic characteristics, traditional cardiovascular risk factors (diabetes mellitus, dyslipidemia, hypertension, obesity, and smoking history), other organ involvement, additional cardiopulmonary parameters, or current medications.

### 3.4. Comparison of Cumulative Manifestations at the Last Visit

Table 2 presents cumulative clinical and cardiopulmonary features at the last visit. Compared with patients without IVCs, patients who developed IVCs had significantly higher cumulative frequencies of digital ulcer, arthritis, atrial fibrillation, and LVEF < 50%. No significant differences were observed between the two groups regarding traditional cardiovascular risk factors, other organ involvement, and additional cardiopulmonary parameters. Patients who developed AF during follow-up received appropriate anticoagulant therapy according to standard clinical indications. No cases of scleroderma renal crisis were identified in this cohort.

### 3.5. Associated Factors for Overall IVCs, CAD, Ischemic Stroke, and Digital Gangrene

Factors associated with the development of overall IVCs and each subtype (CAD, ischemic stroke, and digital gangrene) are summarized in Table 3.

Overall IVCs

Seventeen patients (11.6%) developed IVCs during follow-up. In Univariate Cox regression analysis, ten variables with *p* < 0.10 were included in the multivariate model: baseline (BL) digital pitting, BL digital ulcer, BL traumatic ulcer, BL atrial fibrillation, any atrial fibrillation, any arthritis, BL left ventricular ejection fraction (LVEF) < 50%, any LVEF < 50%, BL pro-BNP, and BL CK. Using multivariate Cox regression with backward selection (removal criterion *p* > 0.1), the following conditions were independently associated with IVCs: BL digital ulcer (adjusted hazard ratio [AHR] 13.70, 95% CI 3.81–49.33, *p* < 0.001), BL traumatic ulcer (AHR 5.51, 95% CI 1.62–18.77, *p* = 0.006), any atrial fibrillation (AHR 8.39, 95% CI 2.25–31.30, *p* = 0.002), BL LVEF < 50% (AHR 13.95, 95% CI 2.23–87.45, *p* = 0.005), any LVEF < 50% (AHR 4.54, 95% CI 1.26–16.32, *p* = 0.020), and elevated BL pro-BNP levels (AHR 1.0004, 95% CI 1.0002–1.0006, *p* < 0.001).

CAD

Five patients (3.4%) developed CAD. Variables associated with CAD in univariate analysis included BL digital ulcer, BL pro-BNP, and BL dyslipidemia (all *p* < 0.05). In the multivariate model with backward elimination (*p* > 0.05 for removal), the following remained independently associated with CAD: BL pro-BNP (AHR 1.0005, 95% CI 1.0000–1.0010, *p* = 0.040) and BL dyslipidemia (AHR 9.74, 95% CI 1.08–87.76, *p* = 0.043).

Ischemic stroke

Four patients (2.7%) developed ischemic stroke. Univariate-associated factors included BL atrial fibrillation, any atrial fibrillation, BL pro-BNP, and BL CK (*p* < 0.05). Multivariate analysis with backward selection identified any atrial fibrillation (AHR 45.26, 95% CI 3.43–596.45, *p* = 0.004) and BL pro-BNP (AHR 1.0007, 95% CI 1.0002–1.0012, *p* = 0.011) as independent factors associated with ischemic stroke.

Digital gangrene

Ten patients (6.8%) developed digital gangrene. Seven variables were associated in univariate analysis (*p* < 0.10): BL digital pitting, BL digital ulcer, BL traumatic ulcer, BL atrial fibrillation, any atrial fibrillation, any arthritis, and any LVEF < 50. After backward selection (*p* > 0.10 for removal), independent predictors were: BL digital ulcer (AHR 7.27, 95% CI 1.66–31.89, *p* = 0.008), BL traumatic ulcer (AHR 13.01, 95% CI 2.79–60.70, *p* = 0.001), and any LVEF < 50% (AHR 9.29, 95% CI 1.73–49.77, *p* = 0.009).

### 3.6. Survival of Patients with Overall IVCs, CAD, Ischemic Stroke, and Digital Gangrene

**Figure 2:**
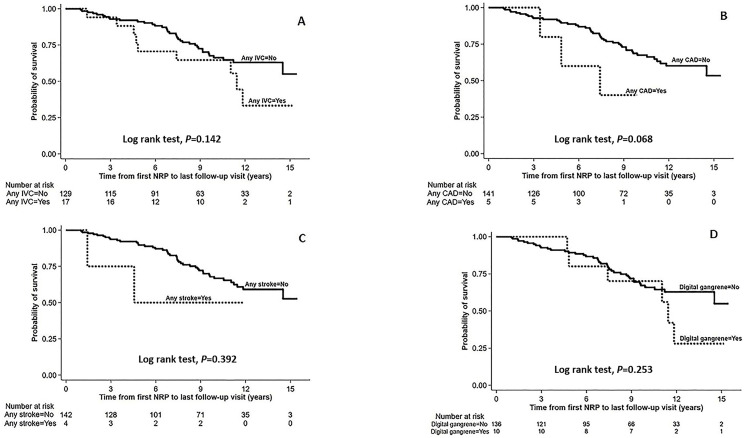
Kaplan–Meier survival curves from the first NRP comparing SSc Patients with and without each complication. (**A**) Overall IVCs, (**B**) CAD, (**C**) ischemic stroke, and (**D**) digital gangrene.

At the end of follow-up, SSc patients with IVCs had significantly higher all-cause mortality than those without IVCs (9 [52.9%] vs. 37 [28.7], *p* = 0.043). Kaplan–Meier survival curves from the first NRP (Figure 2A) showed a trend toward poorer survival in patients with IVCs, although the log-rank test did not reach statistical significance (*p* = 0.142). The incidence rate ratio (IRR) for mortality in the IVC group was 1.81 (95% CI 0.76–3.81, *p* = 0.129), compared with the non-IVC group. Similarly, no significant differences were observed between patients with and without CAD (*p* = 0.068; Figure 2B), ischemic stroke (*p* = 0.392; Figure 2C) or digital gangrene (*p* = 0.253; Figure 2D). However, each subtype showed a trend toward higher mortality: CAD (IRR 2.55, 95% CI 0.50–7.95, *p* = 0.158), ischemic stroke (IRR 1.90, 95% CI 0.22–7.30, *p* = 0.383), and digital gangrene (IRR 1.76, 95% CI 0.61–4.18, *p* = 0.215).

## 4. Discussion

This study represents the first longitudinal inception cohort of patients with early SSc, predominantly with the DcSSc subtype (81.5%) and positive anti-topoisomerase I antibodies (77.4%), which reflects a different genetic background to Western countries [23]. Similar patterns have been reported in a large Thai retrospective SSc cohort, where DcSSc and anti-topoisomerase I antibody positively predominate [24]. The mean disease duration at enrollment was 11.5 months from the first NRP, with a mean follow-up of eight years. The overall incidence rate of IVCs was low at 1.44 per 100 person-years; however, events occurred early, with a median onset of two years after the first NRP.

Independent baseline factors associated with IVCs included digital ulcers, traumatic ulcers, LVEF < 50%, and elevated pro-BNP levels. Notably, pro-BNP levels were significantly higher in patients with IVCs, despite a similar prevalence of echocardiographic PH between groups. Elevated pro-BNP in patients with IVCs may reflect myocardial involvement related to SSc rather than PH alone. During follow-up, atrial fibrillation and reduced LVEF remained independently associated with IVCs. At the end of the study, patients who developed IVCs had significantly higher all-cause mortality than those without IVCs. To our knowledge, no previous study has comprehensively reported the incidence, associated factors, and mortality of IVCs in patients with early SSc.

The incidence rate of CAD in this cohort was 0.40 per 100 person-years, with a median onset of two years, consistent with rates reported in prior large retrospective cohort studies, including Chu et al. [5] (Taiwan: 0.53 per 100 person-years; mean follow-up 4.3 years), Man et al. [8] (UK: 0.44 per 100 person-years; median follow-up 5 years), and Bairkdar et al. [25] (Sweden: 0.75 per person-years; median follow-up 5.2 years). Bairkdar et al. also reported that the risk of acute myocardial infarction (AMI) was highest early after SSc diagnosis [25]. However, differences in study design and the lack of detailed clinical data, such as disease subtype, autoantibody profiles, and disease duration, in these studies limit direct comparison with our cohort. In our cohort, baseline dyslipidemia and elevated pro-BNP were independent factors associated with CAD. Although mortality was higher among patients with CAD, the difference did not reach statistical significance. Prior studies comparing SSc patients with matched general populations found that traditional cardiovascular risk factors—including male gender [6,26], older age [6], hypertension [5,27], diabetes mellitus [5], dyslipidemia [6,27] and SSc-associated PAH [6]—were more common among SSc patients who developed CAD.

The incidence rate of ischemic stroke in our cohort was 0.32 per 100 person-years, with a median onset of 4.5 years. This rate is similar to that reported by Avina-Zubieta et al. [7] (Canada, 0.80 per 100 person-years) and Man et al. [8] (UK, 0.48 per person-years), but lower than those from Taiwan (1.65 per 100 person-years) [28] and the United States (1.53 per 100 person-years, median follow-up five years) [29]. Again, comparisons are limited by incomplete clinical information in prior cohorts. In our study, atrial fibrillation and elevated baseline pro-BNP levels were independently associated with ischemic stroke. Although patients with ischemic stroke showed a trend toward higher mortality, the difference was not statistically significant.

The incidence rate of digital gangrene in our cohort was 0.83 per 100 person-years, with a median time to onset of 1.5 years, which is lower than that reported by Mihai et al. in the EUSTAR cohort (1.94 per 100 person-years) [9]. In that study, patients had a longer mean disease duration (7.9 years), were predominantly female (83.3%), and had a higher prevalence of anti-centromere antibodies (42%). These differences in disease duration, demographic characteristics, subtype distribution, and autoantibody profile may contribute to the higher incidence of digital gangrene observed in the EUSTAR cohort compared with our population.

Independent factors associated with digital gangrene in our cohort included baseline digital ulcer, baseline traumatic ulcer, and LVEF < 50%. Although mortality was higher among patients with digital gangrene, the difference was not statistically significant. Mihai et al. [9] identified older age, digital ulcers, DcSSc subtype, and longer disease duration as risk factors for digital gangrene. Additionally, smoking history, positive anti-centromere and anti-neutrophil cytoplasmic antibodies, antiphospholipid antibodies, and an elevated ESR were also reported as independent predictors by Hui et al. [30], suggesting that inflammatory and macrovascular mechanisms may contribute to the progression of digital gangrene. Variations in incidence and associated factors across studies of IVCs, including CAD, ischemic stroke, and digital gangrene, may reflect differences in study design, population characteristics, and definitions of vascular involvement.

Microvascular and macrovascular involvement in SSc may represent interconnected but temporally distinct processes. Early endothelial injury and fibro-occlusive microangiopathy may drive digital ischemic manifestations, whereas later macrovascular events, including macrovascular coronary and cerebrovascular disease, may reflect cumulative vascular damage, superimposed atherosclerotic risk factors such as dyslipidemia, and evolving cardiac dysfunction. These overlapping mechanisms may explain the differences in timing and clinical patterns of IVCs observed in this cohort.

This study has several limitations. The relatively small sample size and low number of IVC events may have limited statistical power. As a single-center study, the findings may not be generalizable to populations with different disease characteristics. Given the potential regional differences in disease phenotype, these results should be considered hypothesis-generating and interpreted cautiously when extrapolated to broader SSc populations, particularly within the continuum of the early diagnosis of SSc. In addition, the lack of standardized mortality ratio (SMR) data limits the interpretation of survival outcomes. Furthermore, multivariable analyses should be interpreted as exploratory.

Treatment variables were not included in the regression models to minimize indication bias; however, the early management of vascular complications, control of traditional cardiovascular risk factors, and immunosuppressive therapy may have influenced the occurrence of IVCs. Cumulative corticosteroid exposure was not systematically quantified in this cohort; therefore, the potential impact of steroid dose and duration on vascular outcomes cannot be excluded. Nevertheless, long-term corticosteroid doses in this cohort were generally low (mean maintenance dose ≤ 1 mg/day of prednisolone equivalent), consistent with standard clinical practice aimed at minimizing the risk of scleroderma renal crisis. No cases of scleroderma renal crisis were observed during follow-up. In addition, only two of ten patients with digital gangrene underwent angiographic evaluation, both demonstrating evidence of PVD.

Systematic screening for inherited and acquired thrombophilia was not performed. Although APS was clinically excluded, inherited thrombophilic conditions cannot be completely ruled out. However, inherited thrombophilia mutations, such as Factor V Leiden and the prothrombin G20210A mutation, are uncommon in Asian populations and are not routinely assessed in clinical practice [31]. While the baseline ESR was not significantly associated with IVCs in this cohort, C-reactive protein (CRP) levels were not consistently available, limiting the evaluation of inflammatory burden and vascular risk. Finally, vascular dysfunction in SSc may begin during the very early disease stages, including patients fulfilling very early diagnosis of SSc (VEDOSS) criteria [32]. Because nailfold capillaroscopy and anti-RNA polymerase III antibody testing were not routinely available in our center, patients with VEDOSS could not be systematically identified. Further prospective multicenter studies incorporating larger early SSc and VEDOSS populations, standardized vascular assessments, and inflammatory biomarker evaluation are needed to confirm our findings.

Despite these limitations, the study has several strengths. It is the first inception cohort of early SSC patients systematically evaluated for clinical features, biomarkers, and cardiopulmonary involvement, with prospective data collected over eight years. A further strength relates to the definition of the inception time point, which was based on the first NRP rather than the first study visit. Therefore, IVCs detected at baseline evaluation were not considered exclusion criteria if they occurred after early SSc diagnosis. This design reflects the possibility that vascular involvement may precede formal disease recognition in SSc. The longitudinal design allowed consistent and comprehensive follow-up, enhancing reliability. Most prior studies have primarily been prevalent cohort or case–control studies comparing patients with the general population. Our study provides important insights into the incidence, associated factors, and mortality of IVCs, including CAD, ischemic strokes, and digital gangrene in a well-characterized cohort of early SSc patients, the majority of whom had DcSSc with anti-topoisomerase I positivity.

## 5. Conclusions

In this study cohort, the incidence of IVCs was relatively low in early SSc but was associated with increased mortality. Independent factors associated with overall IVCs included digital ulcer, traumatic ulcer, atrial fibrillation, LVEF < 50%, and elevated pro-BNP. Dyslipidemia and elevated pro-BNP were independently associated with CAD, whereas atrial fibrillation and elevated pro-BNP were associated with ischemic stroke. Digital ulcer, traumatic ulcer, and LVEF < 50% were independent factors associated with digital gangrene. These findings highlight clinical features, cardiac parameters, and biomarkers that may be useful in identifying early SSc patients at higher risk of IVCs. Further validation in larger study cohorts is warranted.

## Figures and Tables

**Table 1:** Baseline clinical characteristics, laboratory investigations, and medications in SSc patients with and without IVCs.

Baseline Variables	SSc Without IVCs (n = 129)	SSc with IVCs (n = 17)	*p*-Value
**Demographic**			
Age at SSc diagnosis, yrs.	53.4 ± 9.6	55.1 ± 7.11	0.497
Disease duration 1st NRP to entry, mo.	8 (5, 15.5)	7 (4.5, 20)	0.993
Female	74 (57.4%)	9 (52.9%)	0.729
DcSSc	105 (81.4%)	14 (82.4%)	1.000
Anti-topoisomerase I antibody	100 (77.5%)	13 (76.5%)	1.000
Anti-centromere antibody	8 (6.2%)	3 (17.6%)	0.120
Ever been a smoker	54 (41.9%)	8 (47.1%)	0.684
**Comorbidities**			
Diabetes mellitus	7 (5.4%)	0	1.000
Dyslipidemia	38 (29.5%)	6 (35.3%)	0.622
Hypertension	32 (24.8%)	2 (11.8%)	0.361
Body mass index (kg/mm^2^)	21.1 ± 3.2	21.2 ± 3.40	0.940
**Organ involvement**			
Modified Rodnan skin score	18 (9, 26)	21 (12, 30)	0.267
Raynaud’s phenomenon	114 (88.4%)	15 (88.2%)	1.000
Digital pitting scar	64 (49.6%)	14 (82.4%)	**0.011**
Digital ulcer	4 (3.1%)	6 (35.3%)	**<0.001**
Traumatic ulcer	5 (3.9%)	5 (29.4%)	**0.002**
Arthritis	34 (26.4%)	7 (41.2%)	0.251
Small joint contracture	63 (48.8%)	10 (58.8%)	0.439
Large joint contracture	27 (20.9%)	5 (29.4%)	0.532
Tendon friction rub	11 (8.5%)	3 (17.6%)	0.211
Gastroesophageal reflux disease	55 (42.6%)	5 (29.4%)	0.298
Dysphagia	36 (27.9%)	8 (47.1%)	0.106
**Cardiopulmonary investigations**			
**ECG**			
Atrial fibrillation	0	2 (11.8%)	**0.013**
Conduction defect	21 (16.3%)	3 (17.6%)	1.000
**Echocardiography**			
% LVEF ^a^	68.2 ± 7.0	63.3 ± 13.7	0.171
LVEF < 50%	1 (0.8%)	2 (11.8%)	**0.036**
SPAP, mmHg (n = 100, 16) ^a^	31.8 ± 9.6	35.5 ± 12.2	0.173
TRV, m/s (n = 112, 16) ^a^	2.4 ± 0.4	2.5 ± 0.4	0.758
**Pulmonary function test**			
%pFVC (n = 100, 12) ^a^	73.0 ± 19.0	68.6 ± 18.9	0.551
FEV_1_/FVC (n = 100, 12) ^a^	84.1 ± 15.3	84.3 ± 8.7	0.974
**HRCT**			
Interstitial lung disease	95 (73.6%)	14 (82.4%)	0.562
**Laboratory investigations**			
Hemoglobin, mg/dL ^a^	12.5 ± 1.7	11.9 ± 1.7	0.226
Creatinine, mg/dL ^a^	0.8 ± 0.3	0.8 ± 0.2	0.868
ESR, mm/h ^a^	31 (15, 52)	40 (12, 62.5)	0.563
Creatine kinase, U/L ^b^	139 (79, 285.5)	231 (123.5, 880.5)	**0.046**
Pro-BNP, pg/mL, ^b^	147 (57.5, 321.8)	407 (126.3, 1083.7)	**0.007**
**Current medications**			
Prednisolone	49 (38.0%)	6 (35.3%)	0.830
Cyclophosphamide	31 (24.0%)	3 (17.6%)	0.763
Methotrexate	17 (13.2%)	3 (17.6%)	0.706
Mycophenolate mofetil	5 (3.9%)	1 (5.9%)	0.531
Azathioprine	6 (4.7%)	0	1.000
Calcium channel blocker	106 (82.2%)	15 (88.2%)	0.738
Aspirin	84 (65.1%)	15 (88.2%)	0.055

Values are presented as mean ± SD, median (IQR 1, 3), or n (%). LVEF, left ventricular ejection fraction; SPAP, systolic pulmonary artery pressure; TRV, tricuspid regurgitation velocity; %pFVC, percentage of predicted forced vital capacity; FEV_1_/FVC, forced expiratory volume in one second to forced vital capacity ratio; ESR, erythrocyte sedimentation rate; Pro-BNP, pro-B-type natriuretic peptide. Statistical tests: ^a^, Student-*t* test; ^b^, Mann–Whitney U test. Bold font indicates statistical significance (*p* < 0.05).

**Table 2:** Cumulative clinical and cardiopulmonary characteristics of SSc patients with and without IVCs at the last visit.

Cumulative Variables	SSc Without IVCs (n = 129)	SSc with IVCs (n = 17)	*p*-Value
**Comorbidities**			
Diabetes mellitus	17 (13.2%)	0	0.221
Dyslipidemia	82 (63.6%)	11 (64.7%)	0.927
Hypertension	37 (28.7%)	5 (29.4%)	1.000
**Organ involvement**			
Raynaud’s phenomenon	114 (88.4%)	15 (88.2%)	1.000
Digital pitting	89 (69.0%)	15 (88.2%)	0.153
Digital ulcer	27 (20.9%)	11 (64.7%)	**<0.001**
Traumatic ulcer	38 (29.5%)	9 (52.9%)	0.051
Arthritis	44 (34.1%)	10 (58.8%)	**0.047**
Small joint contracture	85 (65.9%)	15 (88.2%)	0.062
Large joint contracture	49 (38.0%)	8 (47.1%)	0.471
Tendon friction rub	25 (19.4%)	5 (29.4%)	0.345
Gastroesophageal reflux disease	98 (76.0%)	14 (82.4%)	0.763
Dysphagia	63 (48.8%)	11 (64.7%)	0.219
**ECG**			
Atrial fibrillation	7 (5.4%)	4 (23.5%)	**0.025**
Conduction defect	54 (41.9%)	7 (41.2%)	0.957
**Echocardiography**			
Suspected pulmonary hypertension	38 (29.5%)	6 (35.3%)	0.588
LVEF < 50%	11 (8.6%)	5 (29.4%)	**0.024**
**HRCT**			
Interstitial lung disease	106 (82.2%)	15 (88.2%)	0.738

Bold font indicates statistical significance (*p* < 0.05).

**Table 3:** Factors associated with overall IVCs, CAD, ischemic stroke, and digital gangrene.

Variables	Univariate Analysis			Multivariate Cox Regression Analysis		
	HR	95% CI	*p*-Value	AHR	95% CI	*p*-Value
**IVCs**						
BL digital pitting	4.54	1.30–15.80	0.017			
BL digital ulcer	9.01	3.32–24.47	<0.001	13.70	3.81–49.33	**<0.001**
BL traumatic ulcer	5.66	1.99–16.10	0.001	5.51	1.62–18.77	**0.006**
BL atrial fibrillation	15.69	3.42–71.93	<0.001			
Any atrial fibrillation	5.21	1.67–16.23	0.004	8.39	2.25–31.30	**0.002**
Any arthritis	2.47	0.94–6.50	0.067			
BL LVEF < 50%	16.53	3.37–81.02	0.001	13.95	2.23–87.45	**0.005**
Any LVEF < 50%	3.16	1.11–9.01	0.031	4.54	1.26–16.32	**0.020**
BL pro-BNP (pg/mL)	1.0003	1.0001–1.0004	<0.001	1.0004	1.0002–1.0006	**<0.001**
BL creatine kinase (U/L)	1.0005	1.0000–1.0009	0.058			
**CAD**						
BL digital ulcer	7.86	1.31–47.20	0.024			
BL pro-BNP	1.0006	1.0001–1.0010	0.021	1.0005	1.0000–1.0010	**0.040**
BL dyslipidemia	10.63	1.19–95.20	0.035	9.74	1.08–87.76	**0.043**
**Ischemic stroke**						
BL atrial fibrillation	36.68	3.32–405.50	0.003			
Any atrial fibrillation	15.34	2.13–110.69	0.007	45.26	3.43–596.45	**0.004**
BL pro-BNP (pg/mL)	1.0005	1.0001–1.0009	0.005	1.0007	1.0002–1.0012	**0.011**
BL creatine kinase (U/L)	1.0009	1.0003–1.0016	0.006			
**Digital gangrene**						
BL digital pitting	8.62	1.09–68.05	0.041			
BL digital ulcer	11.17	3.12–39.94	<0.001	7.27	1.66–31.89	**0.008**
BL traumatic ulcer	13.13	3.80–45.42	< 0.001	13.01	2.79–60.70	**0.001**
BL atrial fibrillation	8.76	1.09–70.21	0.041			
Any atrial fibrillation	3.88	0.81–18.49	0.089			
Any arthritis	3.87	0.99–14.99	0.050	3.30	0.84–12.95	0.087
Any LVEF < 50%	3.08	0.79–12.01	0.105	9.29	1.73–49.77	**0.009**

HR: hazard ratio; AHR: adjusted hazard ratio; BL: baseline; Any: cumulative manifestations at the last visit. Bold font indicates statistical significance (*p* < 0.05).

## Data Availability

The data presented in this study are available from the corresponding author upon reasonable request in accordance with the regulation of the Institutional Research Ethics Committee.

## References

[1] Gabrielli A., Avvedimento E.V., Krieg T. (2009). Scleroderma. N. Engl. J. Med..

[2] Ngian G.S., Sahhar J., Wicks I.P., Van Doornum S. (2011). Cardiovascular disease in systemic sclerosis--an emerging association?. Arthritis Res. Ther..

[3] Cannarile F., Valentini V., Mirabelli G., Alunno A., Terenzi R., Luccioli F., Gerli R., Bartoloni E. (2015). Cardiovascular disease in systemic sclerosis. Ann. Transl. Med..

[4] Chen I.W., Wang W.T., Lai Y.C., Lin C.M., Liu P.H., Wu S.Z., Hung K.C. (2024). Association between systemic sclerosis and risk of cerebrovascular and cardiovascular disease: A meta-analysis. Sci. Rep..

[5] Chu S.Y., Chen Y.J., Liu C.J., Tseng W.C., Lin M.W., Hwang C.Y., Chen C.C., Lee D.D., Chen T.J., Chang Y.T. (2013). Increased risk of acute myocardial infarction in systemic sclerosis: A nationwide population-based study. Am. J. Med..

[6] Ngian G.S., Sahhar J., Proudman S.M., Stevens W., Wicks I.P., Van Doornum S. (2012). Prevalence of coronary heart disease and cardiovascular risk factors in a national cross-sectional cohort study of systemic sclerosis. Ann. Rheum. Dis..

[7] Aviña-Zubieta J.A., Man A., Yurkovich M., Huang K., Sayre E.C., Choi H.K. (2016). Early Cardiovascular Disease After the Diagnosis of Systemic Sclerosis. Am. J. Med..

[8] Man A., Zhu Y., Zhang Y., Dubreuil M., Rho Y.H., Peloquin C., Simms R.W., Choi H.K. (2013). The risk of cardiovascular disease in systemic sclerosis: A population-based cohort study. Ann. Rheum. Dis..

[9] Mihai C., Distler O., Gheorghiu A.M., Constantin P.I., Dobrota R., Jordan S., Smith V., Hachulla E., Henes J., Siegert E. (2020). Incidence and risk factors for gangrene in patients with systemic sclerosis from the EUSTAR cohort. Rheumatology.

[10] van den Hoogen F., Khanna D., Fransen J., Johnson S.R., Baron M., Tyndall A., Matucci-Cerinic M., Naden R.P., Medsger T.A., Carreira P.E. (2013). 2013 classification criteria for systemic sclerosis: An American college of rheumatology/European league against rheumatism collaborative initiative. Ann. Rheum. Dis..

[11] LeRoy E.C., Black C., Fleischmajer R., Jablonska S., Krieg T., Medsger T.A., Rowell N., Wollheim F. (1988). Scleroderma (systemic sclerosis): Classification, subsets and pathogenesis. J. Rheumatol..

[12] Aletaha D., Neogi T., Silman A.J., Funovits J., Felson D.T., Bingham C.O., Birnbaum N.S., Burmester G.R., Bykerk V.P., Cohen M.D. (2010). 2010 rheumatoid arthritis classification criteria: An American College of Rheumatology/European League Against Rheumatism collaborative initiative. Ann. Rheum. Dis..

[13] Petri M., Orbai A.M., Alarcón G.S., Gordon C., Merrill J.T., Fortin P.R., Bruce I.N., Isenberg D., Wallace D.J., Nived O. (2012). Derivation and validation of the Systemic Lupus International Collaborating Clinics classification criteria for systemic lupus erythematosus. Arthritis Rheum..

[14] Alarcón-Segovia D., Cardiel M.H. (1989). Comparison between 3 diagnostic criteria for mixed connective tissue disease. Study of 593 patients. J. Rheumatol..

[15] Bohan A., Peter J.B. (1975). Polymyositis and dermatomyositis (first of two parts). N. Engl. J. Med..

[16] Shiboski C.H., Shiboski S.C., Seror R., Criswell L.A., Labetoulle M., Lietman T.M., Rasmussen A., Scofield H., Vitali C., Bowman S.J. (2017). 2016 American College of Rheumatology/European League Against Rheumatism Classification Criteria for Primary Sjögren’s Syndrome: A Consensus and Data-Driven Methodology Involving Three International Patient Cohorts. Arthritis Rheumatol..

[17] Grayson P.C., Ponte C., Suppiah R., Robson J.C., Craven A., Judge A., Khalid S., Hutchings A., Luqmani R.A., Watts R.A. (2022). 2022 American College of Rheumatology/European Alliance of Associations for Rheumatology Classification Criteria for Eosinophilic Granulomatosis with Polyangiitis. Ann. Rheum. Dis..

[18] Robson J.C., Grayson P.C., Ponte C., Suppiah R., Craven A., Judge A., Khalid S., Hutchings A., Watts R.A., Merkel P.A. (2022). 2022 American College of Rheumatology/European Alliance of Associations for Rheumatology classification criteria for granulomatosis with polyangiitis. Ann. Rheum. Dis..

[19] Suppiah R., Robson J.C., Grayson P.C., Ponte C., Craven A., Khalid S., Judge A., Hutchings A., Merkel P.A., Luqmani R.A. (2022). 2022 American College of Rheumatology/European Alliance of Associations for Rheumatology classification criteria for microscopic polyangiitis. Ann. Rheum. Dis..

[20] Miyakis S., Lockshin M.D., Atsumi T., Branch D.W., Brey R.L., Cervera R., Derksen R.H., De Groot P.G., Koike T., Meroni P.L. (2006). International consensus statement on an update of the classification criteria for definite antiphospholipid syndrome (APS). J. Thromb. Haemost..

[21] Humbert M., Kovacs G., Hoeper M.M., Badagliacca R., Berger R.M.F., Brida M., Carlsen J., Coats A.J.S., Escribano-Subias P., Ferrari P. (2022). 2022 ESC/ERS Guidelines for the diagnosis and treatment of pulmonary hypertension. Eur. Heart J..

[22] Wangkaew S., Intum J., Prasertwittayakij N., Euathrongchit J. (2022). Elevated baseline serum creatine kinase in Thai early systemic sclerosis patients is associated with high incidence of cardiopulmonary complications and poor survival: An inception cohort study. Clin. Rheumatol..

[23] Louthrenoo W., Kasitanon N., Wichainun R., Wangkaew S., Sukitawut W., Ohnogi Y., Nakaue N., Kuwata S., Takeuchi F. (2013). Association of HLA-DRB1*15:02 and DRB5*01:02 allele with the susceptibility to systemic sclerosis in Thai patients. Rheumatol. Int..

[24] Foocharoen C., Peansukwech U., Mahakkanukrauh A., Suwannaroj S., Pongkulkiat P., Khamphiw P., Nanagara R. (2020). Clinical characteristics and outcomes of 566 Thais with systemic sclerosis: A cohort study. Int. J. Rheum. Dis..

[25] Bairkdar M., Patasova K., Andell P., Holmqvist M. (2025). Increased risk of acute myocardial infarction in Swedish patients with systemic sclerosis: A population-based study. Rheumatol. Adv. Pract..

[26] Yen T.H., Chen Y.W., Hsieh T.Y., Chen Y.M., Huang W.N., Chen Y.H., Chen H.H. (2024). The risk of major adverse cardiovascular events in patients with systemic sclerosis: A nationwide, population-based cohort study. Rheumatology.

[27] Butt S.A., Jeppesen J.L., Torp-Pedersen C., Sam F., Gislason G.H., Jacobsen S., Andersson C. (2019). Cardiovascular Manifestations of Systemic Sclerosis: A Danish Nationwide Cohort Study. J. Am. Heart Assoc..

[28] Chiang C.H., Liu C.J., Huang C.C., Chan W.L., Huang P.H., Chen T.J., Chung C.M., Lin S.J., Chen J.W., Leu H.B. (2013). Systemic sclerosis and risk of ischaemic stroke: A nationwide cohort study. Rheumatology.

[29] Ying D., Gianfrancesco M.A., Trupin L., Yazdany J., Greidinger E.L., Schmajuk G. (2020). Increased Risk of Ischemic Stroke in Systemic Sclerosis: A National Cohort Study of US Veterans. J. Rheumatol..

[30] Hui M., Zhou J., Li M., Wang Q., Zhao J., Hou Y., Xu D., Zeng X. (2024). Digital gangrene in systemic sclerosis patients: Not only due to the microvascular disease. Clin. Rheumatol..

[31] Boonyawat K., Angchaisuksiri P. (2022). Thrombosis and anticoagulation: Clinical issues of special importance to hematologists who practice in Asia. Hematol. Am. Soc. Hematol. Educ. Program.

[32] Avouac J., Fransen J., Walker U.A., Riccieri V., Smith V., Muller C., Miniati I., Tarner I.H., Randone S.B., Cutolo M. (2011). Preliminary criteria for the very early diagnosis of systemic sclerosis: Results of a Delphi Consensus Study from EULAR Scleroderma Trials and Research Group. Ann. Rheum. Dis..

